# The Impact of Generative AI Dependence on University Students’ Creativity: The Mediating Role of Critical Thinking and the Moderating Role of Thinking Orientation

**DOI:** 10.3390/jintelligence14080168

**Published:** 2026-08-01

**Authors:** Fang Yuan, Chunlei Liu, Jiaqin Yang

**Affiliations:** 1 Faculty of Education, Qufu Normal University, Qufu 273165, China; mathyf@qfnu.edu.cn; 2 School of Mathematical Sciences, Qufu Normal University, Qufu 273165, China; 3 School of Psychology, Qufu Normal University, Qufu 273165, China; yangjq2015@qfnu.edu.cn

**Keywords:** generative AI dependence, creativity, critical thinking, thinking orientation, moderated mediation

## Abstract

The rapid proliferation of generative artificial intelligence (AI) has raised concerns about its impact on creativity, yet mechanisms and boundary conditions remain underexplored. Using one survey and two experiments, we investigated how AI dependence affects university students’ creativity. Study 1 (cross-sectional) found AI dependence was negatively related to creativity, fully mediated by critical thinking. Study 2 (between-subjects experiment) experimentally demonstrated that high AI dependence was associated with lower creativity than low dependence, with critical thinking again mediating. Study 3 employed a 2 × 2 design with thinking orientation as a moderator in a first-stage moderated mediation model. Thinking orientation moderated the “AI dependence → critical thinking → creativity” pathway: the indirect effect was significant under an outcome-oriented condition but non-significant under a process-oriented condition; the index of moderated mediation was 0.22. These findings indicate that critical thinking is a central pathway through which AI dependence undermines creativity, and process-oriented thinking buffers this negative impact by safeguarding critical thinking. The study provides theoretical foundations and intervention directions for the judicious use of AI in education.

## 1. Introduction

Generative artificial intelligence (AI) tools, such as ChatGPT and DeepSeek, are permeating academic research and knowledge production at an unprecedented pace. University students, as learners and the backbone of future innovation, are among the most active users of generative AI. However, whether generative AI use facilitates or inhibits users’ creativity remains an unresolved question (E. Zhou & Lee, 2024).

Some researchers emphasize the enabling effects of AI. Boussioux et al. (2024) found that AI can significantly enhance the quantity and quality of creative outputs; Chandrasekera et al. (2024) also noted that AI can support creativity in early design processes. In tension with this view, Doshi and Hauser (2024) demonstrated through a large-scale experiment that although AI raises the average level of individual creative output, it reduces the diversity of creative content. Zhang et al. (2024) directly proposed the concept of “AI dependency,” arguing that when individuals become overly dependent on AI, their autonomous learning capacity and independent thinking quality may be undermined. Li and Ganapathy (2026) found that AI-assisted learning significantly influences critical thinking among Chinese English majors, suggesting that it is the manner of AI use, rather than frequency per se, that critically shapes cognitive quality. Cui et al. (2026) further revealed that dependence on AI has dual effects on employees’ creativity—a finding that provides direct theoretical precedent for the present study’s examination of thinking orientation as a moderator.

The above divergence implies that the AI–creativity relationship may not be a simple linear one but rather involves complex mediating mechanisms and boundary conditions. The present study seeks to advance understanding of this issue at two levels. First, we aim to reveal the mediating role of critical thinking. According to Sternberg’s (2003) integrative theory of creativity, cognitive abilities constitute the core components of creativity. Among these, critical thinking is essential for the analysis, evaluation, and integration of information (Dwyer et al., 2025). If AI dependence weakens individuals’ critical thinking—for instance, by causing individuals to habitually accept AI outputs rather than actively question and reflect upon them—the decline in creativity could be explained through this pathway. Second, we explore the moderating role of thinking orientation. Research on mental simulation (Escalas & Luce, 2004; Pham & Taylor, 1999) distinguishes between an outcome orientation (focusing on final outputs) and a process orientation (focusing on the learning experience). In AI-assisted contexts, outcome-oriented individuals may be more inclined to forgo deep thinking and directly adopt AI suggestions, whereas process-oriented individuals may sustain greater cognitive engagement and critical processing. Recent research suggests that critical thinking disposition can indirectly promote scientific creativity through creative self-efficacy (Qiang et al., 2020), highlighting the critical role of cognitive mindsets in moderating creative cognition pathways. Accordingly, thinking orientation may serve as a key boundary condition moderating the effects of AI dependence.

The present research seeks to fill two specific gaps in the current literature. First, although prior studies have separately documented negative links between AI dependence and cognitive outcomes (Zhang et al., 2024) and positive links between critical thinking and creativity (Park et al., 2026; Dwyer et al., 2025), no study to date has integrated these strands into a unified moderated mediation model that explains how and under what conditions AI dependence impairs creativity. By positioning critical thinking as the mediating mechanism and thinking orientation as the boundary condition, we offer a mechanistic account that moves beyond the dichotomous “positive versus negative” debate in the AI–creativity literature. Second, although Cui et al. (2026) identified dual effects of AI dependence on employee creativity, their correlational design could not establish causality or isolate the cognitive pathway. The present three-study design is therefore deliberately incremental: Study 1 establishes the basic correlational model and tests the mediation hypothesis in a naturalistic setting; Study 2 experimentally manipulates AI dependence to confirm causal direction and isolate critical thinking as the mediator while ruling out self-efficacy as an alternative explanation; and Study 3 introduces an experimental moderator to construct a complete first-stage moderated mediation model. This triangulation across methods—correlational survey plus two experiments—provides converging evidence for the proposed mechanism and its boundary conditions, addressing both the “what” and the “when” of AI dependence effects on creativity.

**Figure 1:**
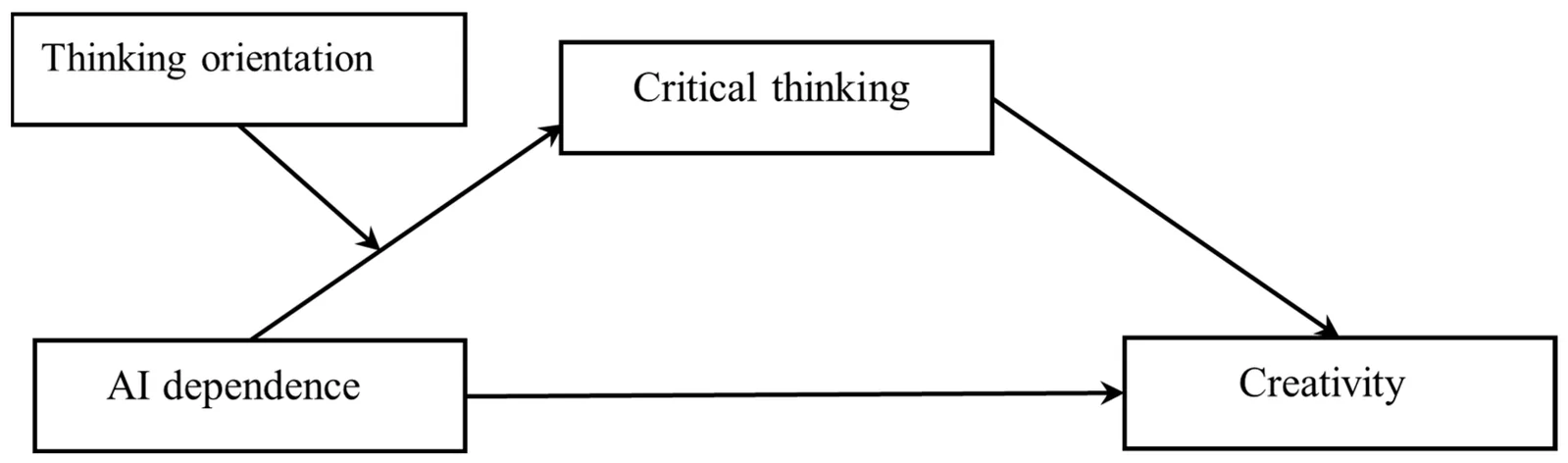
The theoretical framework of this study.

To systematically test these hypotheses, we designed three progressive studies: Study 1, a cross-sectional survey, preliminarily establishes the relational model among AI dependence, critical thinking, and creativity; Study 2, a single-factor experiment manipulating AI dependence levels, tests the causal direction and the hypothesised mediating role of critical thinking; Study 3 further incorporates thinking orientation as a moderator to construct a complete moderated mediation model, revealing the boundary conditions of AI dependence’s impact on creativity. The overall theoretical framework is presented in Figure 1.

## 2. Study 1: Cross-Sectional Survey

### 2.1. Objective

Study 1 aimed to preliminarily examine the relationship between generative AI dependence and university students’ creativity, as well as the mediating role of critical thinking, through a cross-sectional survey design.

### 2.2. Method

**Participants.** A total of 142 university students (including graduate students) with generative AI usage experience were recruited via online platforms (63 males, 79 females; mean age = 24.32, SD = 2.15) from Qufu Normal University, a comprehensive research-intensive university in Shandong, China. The institution enrolls approximately 30,000 undergraduate and approximately 5000 graduate students across programmes in the social sciences, natural sciences, engineering and technology, and arts and humanities. At the time of data collection, the university had no institution-wide policy prohibiting generative AI use in coursework; individual instructors set their own guidelines, ranging from unrestricted use to complete prohibition. Participants were recruited via campus-wide online platforms (63 males, 79 females; mean age = 24.32, SD = 2.15). The sample spanned the four disciplinary clusters noted above, with roughly equal representation across colleges.

**Sample size justification.** An a priori power analysis was conducted using G*Power 3.1 for a bivariate correlation analysis. Assuming a medium effect size (ρ = 0.30), α = 0.05 (two-tailed), and statistical power of 0.80, the required minimum sample size was 84 participants. The actual sample of 142 participants substantially exceeded this threshold, ensuring adequate statistical power for the planned correlational and mediation analyses.

**Measures.** The Generative AI Dependence Scale developed by Tang et al. (2023) was used (3 items, α = 0.79, e.g., “I depend on generative AI to handle or assist with academic research/study tasks”), rated on a 5-point Likert scale. Creative thinking was measured using a general creativity scale (adapted from Akpur, 2020; 6 items, α = 0.87). Sample items include: “I am able to find new uses for existing products or ideas” and “I enjoy thinking of new ways to solve problems.” Responses were rated on a 5-point Likert scale ranging from 1 (almost never) to 5 (always). This scale captures self-perceived creative capacity across domains rather than performance on a specific creative task. Critical thinking was assessed with the Chinese Critical Thinking Structure Scale developed by Hou et al. (2022) (17 items, α = 0.85, comprising three dimensions: analyticity, open-mindedness, and systematicity). All scales used 5-point Likert scoring. The complete items of all scales are provided in the Supplementary Materials.

### 2.3. Results and Analysis

**Common method bias test.** Harman’s single-factor test was conducted. An unrotated exploratory factor analysis extracted seven factors with eigenvalues greater than 1, with the first factor accounting for 24.37% of the total variance, well below the 40% threshold, indicating that common method bias was not a serious concern. Notwithstanding Harman’s single-factor test result, we acknowledge that all three key constructs—AI dependence, critical thinking, and creativity—were measured via self-report in Study 1, which raises the possibility of common method variance (CMV). Although the single-factor extraction accounted for only 24.37% of the variance, well below the 40% threshold, CMV can inflate observed correlations through shared rater biases, consistency motifs, or transient mood states (Podsakoff et al., 2003). The cross-sectional nature of Study 1 further precludes causal inference. These limitations were explicitly addressed in the subsequent experimental studies: Study 2 manipulated AI dependence experimentally and employed independent raters for creativity assessment (the Consensual Assessment Technique), thereby breaking the single-source dependency; Study 3 additionally introduced an experimental moderator. The convergence of findings across different methodological approaches strengthens confidence that the observed mediation pathway is not an artefact of CMV, but readers should remain cautious when interpreting the effect sizes from Study 1.

**Descriptive statistics and correlations.** The means, standard deviations, and correlation coefficients for all variables are presented in Table 1. Generative AI dependence was significantly negatively correlated with creativity (r = −0.20, *p* = 0.019) and with critical thinking (r = −0.44, *p* < 0.001); critical thinking was significantly positively correlated with creativity (r = 0.50, *p* < 0.001).

**Figure 2:**
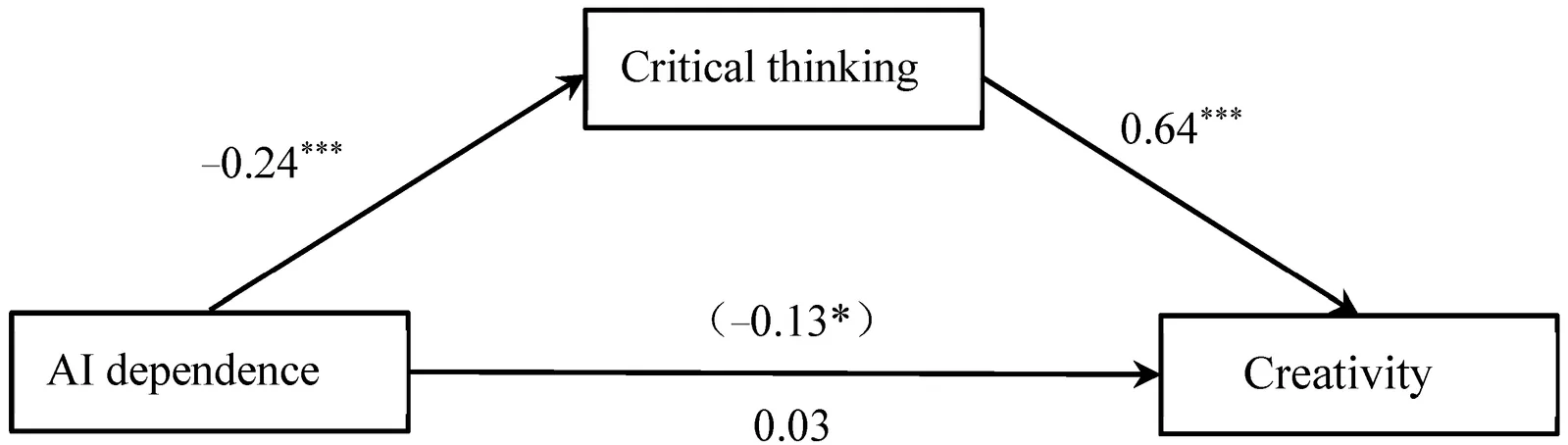
The mediating path of critical thinking between generative AI dependence and creativity (Study 1). Note. * *p* < 0.05, *** *p* < 0.001.

**Mediation analysis.** The PROCESS macro (Model 4; Hayes, 2018) with 5000 bootstrap resamples was used to test the mediating effect of critical thinking (see Figure 2). Results indicated that the total effect of AI dependence on creativity was significant (B = −0.22, SE = 0.09, *p* = 0.019, 95% CI = [−0.40, −0.04]). After including critical thinking, the direct effect became non-significant (B = −0.07, SE = 0.11, *p* = 0.52), while the indirect effect was significant (indirect effect = −0.15, Boot SE = 0.06, 95% CI = [−0.34, −0.10]), indicating that critical thinking fully mediated the impact of AI dependence on creativity.

### 2.4. Summary

Study 1 provided preliminary evidence for a negative relationship between generative AI dependence and creativity and provided preliminary support for critical thinking as a mediating mechanism. However, the cross-sectional design limits causal inference. Study 2 addressed this limitation by experimentally manipulating AI dependence levels to further establish causality.

## 3. Study 2: AI Dependence Manipulation Experiment

### 3.1. Objective

Study 2 employed a single-factor between-subjects experimental design, manipulating generative AI dependence levels (high-dependence vs. low-dependence groups), to test the causal effect of AI dependence on creativity and the mediating role of critical thinking.

### 3.2. Method

**Participants.** A total of 132 university students (including graduate students; 51 males, 81 females) were recruited and randomly assigned to either the high-dependence group (*n* = 66) or the low-dependence group (*n* = 66). The two groups did not differ significantly in age, gender, or disciplinary background.

**Sample size justification.** An a priori power analysis was conducted using G*Power 3.1 for an independent-samples *t*-test. Assuming a medium effect size (Cohen’s d = 0.50), α = 0.05 (two-tailed), and statistical power of 0.80, the required minimum sample size was 102 participants (51 per group). The actual sample of 132 participants (66 per group) exceeded this minimum, ensuring adequate power for detecting the hypothesised group differences.

It is important to note that this manipulation reflects a combination of reliance (mandatory use of AI), trust (statements that AI answers are optimal), and reduced personal contribution (direct adoption of AI outputs), rather than a pure manipulation of trait-like AI dependence or usage frequency alone. We therefore interpret the “high-dependence” condition as an operationalisation of state dependence—a temporary psychological state characterised by high reliance on and trust in AI-generated content. While this design does not isolate the unique contribution of each component, it captures the holistic experience of AI dependence as it manifests in educational contexts. Recent research on AI overreliance similarly operationalises dependence through a combination of trust induction and reduced autonomy (Klingbeil et al., 2024), suggesting that our approach is consistent with the emerging experimental literature in this domain.

**Experimental design and procedure.** The experiment was conducted in two phases. In Phase 1, the priming task, the high-dependence group was instructed to strictly use ChatGPT-generated answers for three tasks (mathematical calculation, text summarisation, and English translation), with instructions emphasising that “the answers provided by the AI are optimal”; the low-dependence group completed the same tasks independently without using any external tools, including calculators, translation applications, search engines, or AI assistance. Participants were explicitly informed of this prohibition at the beginning of Phase 1 and again before the creativity task in Phase 2. Compliance was monitored by having an experimenter present in the laboratory room throughout the session and by requiring participants to complete all tasks in a restricted computer environment (full-screen browser lockdown) that prevented access to unauthorised websites or applications. In Phase 2, the creativity task, participants in both groups used ChatGPT to participate in a “creative competition.” The task required generating as many innovative uses as possible for everyday objects (“tennis racket” and “water pipe”), following the Alternative Uses Task paradigm (AUT; Guilford, 1967). The high-dependence group was instructed to directly adopt the creative solutions generated by ChatGPT, whereas the low-dependence group was instructed to use AI only as a supplementary reference and to independently complete their final solutions.

**Measures.** The AI dependence manipulation check employed the 3-item scale by Tang et al. (2023). Critical thinking was measured as in Study 1. Creativity was rated using the Consensual Assessment Technique (CAT; Amabile, 1982). Three trained raters independently scored each response on three dimensions—novelty, appropriateness, and fluency—using a 7-point Likert scale anchored at 1 (“not at all creative/not at all appropriate/not at all fluent”) and 7 (“extremely creative/extremely appropriate/extremely fluent”). Raters were three graduate students in psychology who had completed a 2 h calibration session involving discussion of anchor responses and practice rating of 20 pilot responses that were not part of the main study. Intraclass correlation coefficient (ICC) across the three raters was 0.86, indicating excellent inter-rater reliability. Importantly, raters were blind to participants’ experimental condition; response sheets were presented in random order with all condition-identifying information removed. The mean of the three raters’ scores was used as the creativity index. In addition, self-efficacy (adapted from Wang & Chuang, 2024; 3 items, α = 0.76) was measured as an alternative mediator.

### 3.3. Results

**Manipulation check.** The AI dependence manipulation check score in the high-dependence group (M = 4.39, SD = 0.84) was significantly higher than that in the low-dependence group (M = 3.82, SD = 1.07), *t*(130) = −3.45, *p* = 0.001, Cohen’s d = −0.60, confirming that the manipulation was effective.

**Creativity performance.** The low-dependence group scored significantly higher on creativity (M = 5.20, SD = 0.53) than the high-dependence group (M = 4.78, SD = 0.66), *t*(130) = 4.03, *p* < 0.001, Cohen’s d = 0.70, a medium-to-large effect size.

**Critical thinking.** The low-dependence group scored significantly higher on critical thinking (M = 3.17, SD = 0.28) than the high-dependence group (M = 2.78, SD = 0.29), *t*(130) = 7.94, *p* < 0.001, Cohen’s d = 1.37, a very large effect size. Descriptive statistics and between-group comparisons for all variables are presented in Table 2.

**Figure 3:**
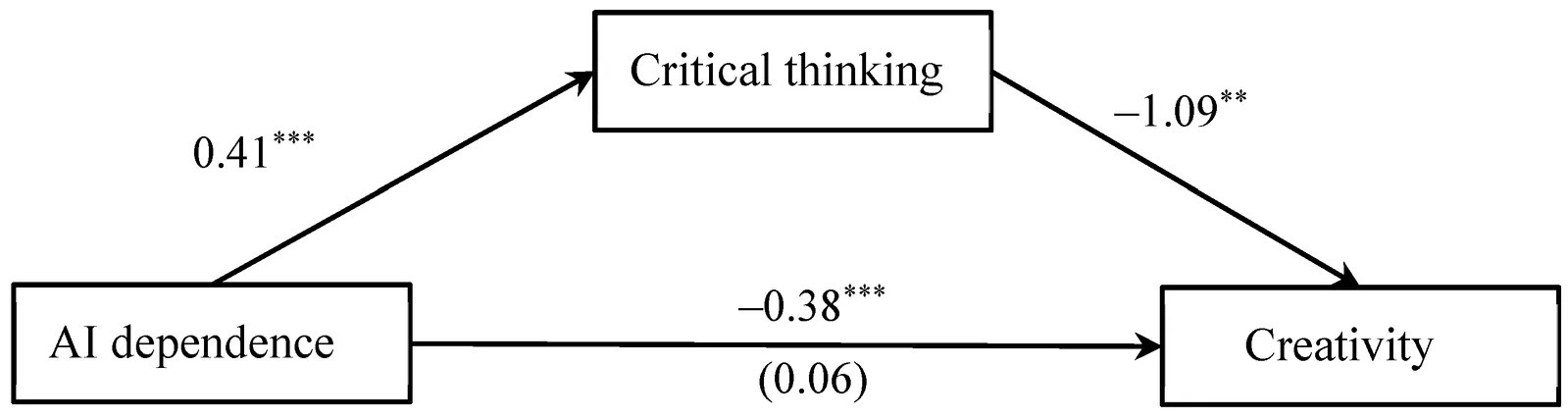
The mediating path of critical thinking between generative AI dependence and creativity (Study 2). Note. ** *p* < 0.01, *** *p* < 0.001.

**Mediation analysis.** The bootstrap method (5000 resamples) was used to test the mediating effect of critical thinking (see Figure 3). Results showed that the direct path from AI dependence to critical thinking was significant (path a: β = −0.40, *p* < 0.001); after controlling for AI dependence, the path from critical thinking to creativity was β = 1.01. The total effect c = −0.42, direct effect c’ = −0.02, indirect effect ab = −0.40, bootstrap 95% CI = [−0.70, −0.14], not containing zero, indicating that the mediating effect of critical thinking was significant and consistent with a full-mediation pattern (c’ approaching zero). It is important to note, however, that because critical thinking was measured rather than manipulated in this experimental design, we cannot definitively establish that critical thinking causes the reduction in creativity; rather, the data are consistent with the hypothesised causal chain (AI dependence → reduced critical thinking → reduced creativity), and the experimental evidence supports this pathway without ruling out all alternative explanations. As an alternative explanation, the mediating effect of self-efficacy was non-significant (indirect effect = −0.12, 95% CI = [−0.40, 0.16], containing zero), ruling out self-efficacy as an alternative mediator.

### 3.4. Summary

Study 2 provided experimental evidence for the impairing effect of AI dependence on creativity and again supported critical thinking as the primary mediating pathway, ruling out the alternative explanation of self-efficacy. However, this study left unanswered a crucial question: Are there boundary conditions under which the negative effects of AI dependence can be moderated? In other words, do certain usage styles buffer or block the detrimental impact of AI dependence on creativity?

## 4. Study 3: The Moderating Role of Thinking Orientation

### 4.1. Objective

Study 2 confirmed the mediating role of critical thinking in the AI dependence–creativity relationship, but individual differences in thinking styles may further moderate this pathway. Study 3 aimed to test the moderating role of thinking orientation (outcome-oriented vs. process-oriented) in the “AI dependence → critical thinking → creativity” mediation pathway, constructing a first-stage moderated mediation model. We specifically hypothesised that an outcome orientation would amplify the impairing effect of AI dependence on critical thinking, whereas a process orientation would buffer this effect.

### 4.2. Method

**Participants.** A total of 160 university students (including graduate students; 68 males, 92 females) were recruited and randomly assigned to one of four experimental conditions: low AI-dependence/outcome-oriented (*n* = 41), low AI-dependence/process-oriented (*n* = 39), high AI-dependence/outcome-oriented (*n* = 40), and high AI-dependence/process-oriented (*n* = 40).

**Sample size justification.** An a priori power analysis was conducted using G*Power 3.1 for a 2 × 2 between-subjects factorial ANOVA. Assuming a medium effect size (f = 0.25), α = 0.05, and statistical power of 0.80, the required minimum sample size was 128 participants (32 per cell). The actual sample of 160 participants (40 per cell) exceeded this minimum, ensuring adequate power for detecting the hypothesised main and interaction effects.

**Experimental design.** The experiment was conducted in three phases. Phase 1 involved the AI dependence manipulation, using a modified trust game paradigm (Klingbeil et al., 2024): participants faced a series of decision-making problems; the high-dependence group was encouraged to fully trust the AI’s investment advice, while the low-dependence group was instructed to exercise autonomous judgment. Phase 2 involved the thinking orientation manipulation, following a behavioural priming paradigm (Dai et al., 2023): the outcome-oriented group read and rated five biased statements emphasising that “outcomes are more important than the process” (e.g., “A good result is more important than effort in the process”); the process-oriented group read and rated statements emphasising that “the process is more important than outcomes” (e.g., “Learning and growth in the process are more valuable than the final result”). Phase 3 was the creativity task, identical to the AUT used in Study 2. Manipulation checks and dependent variable measures were the same as in the previous studies.

### 4.3. Results

**Manipulation checks.** The AI dependence manipulation was effective: the high-dependence group’s AI dependence check score (M = 6.06, SD = 0.79) was significantly higher than that of the low-dependence group (M = 4.95, SD = 1.70), *t*(158) = 5.32, *p* < 0.001, Cohen’s d = 0.84. The thinking orientation manipulation was also effective: the outcome-oriented group’s thinking orientation check score (M = 4.53, SD = 1.77) was significantly higher than that of the process-oriented group (M = 3.61, SD = 1.87), *t*(158) = 3.18, *p* = 0.002, Cohen’s d = 0.50.

**The Moderating Role of Outcome/Process Orientation**

**(1) Critical Thinking**

A hierarchical moderated regression (Table 3) showed that in Step 1, covariates explained 5% of variance (*p* = 0.175). In Step 2, process orientation significantly predicted higher critical thinking (B = 0.28, *p* < 0.001), whereas AI dependence did not (B = −0.10, *p* = 0.160). In Step 3, the AI dependence × thinking orientation interaction was significant (B = 0.34, *p* < 0.01), adding 4% incremental variance.

To facilitate interpretation of the interaction pattern, we plotted the simple slopes of AI dependence on critical thinking at each level of thinking orientation. Under outcome orientation, the slope was steep and negative: participants in the high-dependence condition scored substantially lower on critical thinking than those in the low-dependence condition. Under process orientation, the slope flattened: high-dependence participants actually scored slightly (though non-significantly) higher on critical thinking than low-dependence participants, indicating that a process-oriented mindset effectively neutralised the cognitive cost of AI dependence. The crossover pattern of the interaction underscores that process orientation does not merely attenuate the negative effect of AI dependence; it appears to reverse the direction of the relationship within the experimental context.

Simple-effects tests revealed that under outcome orientation, high-dependence participants scored lower on critical thinking (M = 3.16, SD = 0.47) than low-dependence participants (M = 3.43, SD = 0.36), *t*(77) = 2.84, *p* = 0.006. Under process orientation, this difference disappeared, *t*(77) = −0.78, *p* = 0.437 (M_high = 3.61, SD = 0.46; M_low = 3.53, SD = 0.35).

**(2) Creativity**

A parallel hierarchical regression on expert-rated creativity (Table 4) showed that in Step 2, AI dependence had a strong negative effect (B = −1.35, *p* < 0.001) and process orientation had a strong positive effect (B = 1.20, *p* < 0.001). In Step 3, the interaction was significant (B = 0.39, *p* < 0.05), adding 1% incremental variance.

Simple-effects tests showed that under outcome orientation, low-dependence participants significantly outperformed high-dependence participants on creativity (M_low = 4.52, SD = 1.07 vs. M_high = 2.99, SD = 0.28), *t*(77) = 8.87, *p* < 0.001, Cohen’s d = 1.95. Under process orientation, the negative effect was substantially attenuated but remained significant (M_low = 5.55, SD = 0.19 vs. M_high = 4.40, SD = 0.25), *t*(77) = 22.70, *p* < 0.001, Cohen’s d = 5.09.

**(3) Moderated mediation model.** The bootstrap method (5000 resamples) was used to test the first-stage moderated mediation model (AI manipulation → critical thinking → creativity, with orientation manipulation moderating the first-stage path). Regression analyses showed that the main effects of AI dependence (a_1_ = −0.27) and thinking orientation (a_2_ = 0.10) on critical thinking, as well as their interaction (a_3_ = 0.34), were all significant; the effect of critical thinking on creativity was b = 0.66.

Conditional indirect effect analysis (see Table 5) revealed that under the outcome-oriented condition (W = 0), the indirect effect of AI dependence on creativity via critical thinking was β = −0.18, bootstrap 95% CI = [−0.33, −0.05], CI not containing zero, indicating a significant mediation effect; under the process-oriented condition (W = 1), this indirect effect was β = 0.05, bootstrap 95% CI = [−0.07, 0.17], CI containing zero, indicating a non-significant mediation effect. The index of moderated mediation (IMM = a_3_ × b) was 0.22, bootstrap 95% CI = [0.05, 0.43], not containing zero, confirming that the moderated mediation effect was significant. Conceptually, the IMM represents the difference in the indirect effect of AI dependence on creativity (via critical thinking) between the outcome-oriented and process-oriented conditions. A positive IMM of 0.22 indicates that the indirect effect was 0.22 units less negative (i.e., more buffered) under process orientation than under outcome orientation. In sum, thinking orientation significantly moderates the entire “AI dependence → critical thinking → creativity” mediation pathway: an outcome orientation activates this pathway (higher AI dependence → lower critical thinking → lower creativity), whereas a process orientation renders this pathway non-significant.

### 4.4. Summary

Study 3 revealed a key boundary condition in the impact of AI dependence on creativity. An outcome orientation amplifies, while a process orientation buffers, the negative effects of AI dependence, with this moderation operating through the critical thinking mediation pathway. This finding provides a direct theoretical basis for designing targeted educational interventions.

## 5. General Discussion

### 5.1. Key Findings and Theoretical Contributions

Through one survey and two experiments, the present research systematically elucidated the mechanism by which generative AI dependence affects university students’ creativity, making three principal contributions.

First, this study clarified the cognitive mechanism through which AI dependence undermines creativity. The existing literature presents opposing views on the AI–creativity relationship—positive (Boussioux et al., 2024; Chandrasekera et al., 2024) and negative (Doshi & Hauser, 2024; Zhang et al., 2024). Recent research has further revealed the complexity of this relationship: a recent meta-analysis found a significant yet complex association between creativity and critical thinking (Park et al., 2026); Stadler et al. (2024) showed that over-reliance on large language models reduces deep processing, thereby compromising cognitive quality. The present study suggests that the divergence in prior findings may stem from insufficient attention to mediating mechanisms: when AI dependence impairs critical thinking (as evidenced by the very large effect size of Cohen’s d = 1.37 for the critical thinking group difference in Study 2 and d = 1.95 for the creativity group difference under the outcome-oriented condition in Study 3), a decline in creativity follows. Studies 1 and 2 provided converging evidence for this mediation pathway at both correlational and experimental levels, and Study 2 additionally ruled out self-efficacy as an alternative mediator. These findings are consistent with the interpretation that critical thinking plays a central mediating role in the AI dependence–creativity relationship, although the experimental design (measuring rather than manipulating critical thinking) means that causal mediation is inferred rather than definitively established. This finding aligns closely with the integrative theory of creativity (Sternberg, 2003)—as a component of “creativity-relevant processes,” impairment in critical thinking is likely to compromise overall creative performance.

Second, the study identified thinking orientation as a salient boundary condition and constructed a complete moderated mediation model. Study 3 demonstrated that process-oriented thinking buffers the negative effects across the entire “AI dependence → critical thinking → creativity” causal chain, while an outcome orientation exacerbates them. This finding echoes the work of Cui et al. (2026), who found that dependence on AI has dual effects on employee creativity. Under the outcome-oriented condition, the creativity of the high AI-dependence group dropped to M = 2.99, whereas under the process-oriented condition, creativity in the same “high-dependence” state remained at M = 4.40—a protective effect that is not only statistically significant but also practically substantial within this specific experimental paradigm. Whether this buffering effect generalises to more complex, authentic creative tasks (e.g., essay writing, research proposal design) awaits further investigation. At the theoretical level, this finding is consistent with research on process-outcome mental simulation (Taylor et al., 1998; Pham & Taylor, 1999): a process orientation prompts individuals to maintain cognitive engagement and reflective processing, thereby resisting the erosion of deep thinking caused by AI dependence. More importantly, the moderated mediation model established in the present study offers an integrative mechanistic framework for understanding why the manner of AI use makes a difference.

Third, the study advances creativity research with a new agenda for the AI era. Traditional creativity research has predominantly focused on the roles of individual traits (Guilford, 1950), cognitive processes (Mumford et al., 2002), or social environments (Sternberg, 2003). In the age of AI, E. Zhou and Lee (2024) pioneered the exploration of the interaction between generative AI and human creativity from an artistic creation perspective, and Y. Zhou et al. (2026) further revealed a potential “creative scar” effect of generative AI use—where individual creativity fails to sustain while homogeneity keeps climbing. By focusing on the emerging context of human–AI interaction, the present study reveals how AI tools indirectly shape creative output by influencing users’ cognitive qualities (critical thinking). This perspective injects a new dimension of technological mediation into creativity research and resonates with recent claims that it is the manner of AI use, rather than the frequency of use, that determines cognitive consequences (Li & Ganapathy, 2026).

### 5.2. Practical Implications

The findings of this study carry direct implications for educational practice.

For learners, the critical issue is not whether to use AI but how to use it. The risk of over-reliance on AI lies in its potential to inadvertently “outsource” critical thinking—when individuals habitually accept AI outputs without questioning or filtering, creativity quietly diminishes (Study 2). Learners should maintain a critical stance toward AI-generated content, treating AI as a starting point for thinking rather than an endpoint.

For educators, the significant protective effect of process orientation in Study 3 suggests that “critical thinking + AI use” should be treated as an integrated educational objective. However, because Study 3 manipulated thinking orientation through a brief situational prime, its immediate effect may not be durable. Sustained, curriculum-level interventions would likely be needed to develop stable process-oriented habits in students. We offer concrete examples at three instructional levels: At the course level, instructors could redesign the syllabus to frame AI as a critical dialogue partner rather than an answer source. For example, in a research methods course, students might be required to submit an “AI critique log” alongside their final proposal: after using AI to generate a literature-review outline, students must annotate each AI-suggested theme with one supporting reference they verified independently and one counterargument they identified. This maps directly onto the process-oriented emphasis in Study 3—specifically, the instruction to value “learning and growth in the process”—by foregrounding cognitive effort over final output quality. At the assignment level, process-oriented instructions can be embedded in task prompts. Rather than asking students to “write an essay using AI assistance,” instructors could specify: “Use ChatGPT to generate three alternative thesis statements for your essay. For each, evaluate its logical consistency, empirical support, and originality in a 200-word justification. Then revise the strongest statement into your final thesis.” This procedure mirrors the protective mechanism observed in Study 3’s process-oriented condition: it forces students to maintain critical engagement (evaluating, comparing, revising) rather than directly adopting AI outputs, thereby preserving the cognitive habits that AI dependence otherwise erodes. At the classroom activity level, brief in-class exercises can reinforce process-oriented habits. For instance, a 15 min “AI Devil’s Advocate” activity could ask students to present an AI-generated solution to a problem and then spend five minutes identifying its hidden assumptions, potential biases, or logical gaps before discussing improvements. Such activities instantiate the “questioning–analysis–reconstruction” cycle that our findings identify as the core protective mechanism. Importantly, Study 3 reveals that the intervention effect does not require complete AI abstinence—under high AI-dependence conditions, merely shifting the usage style from outcome-oriented to process-oriented produced a significant rebound in creativity. This suggests that educators should aim for reflective integration rather than prohibition.

For AI tool designers, the findings suggest optimising interaction design to promote reflective use. For example, embedding guided questions after output delivery (e.g., “Do you agree with this conclusion? If not, please explain why”), or providing multi-solution comparison features rather than a single output, could help sustain users’ critical engagement (Longoni et al., 2019).

### 5.3. Limitations and Future Directions

This study has several limitations, each of which points to a concrete direction for future research. First, the cross-sectional design of Study 1 limits causal inference. Future research should employ longitudinal designs (e.g., three-wave panel studies across a semester) to trace the temporal ordering of AI dependence, critical thinking, and creativity, and to test whether changes in AI dependence precede declines in critical thinking and creativity over time. Second, the experimental manipulations in Studies 2 and 3 conflate AI dependence with reduced autonomy, increased trust, and lower cognitive engagement. We cannot rule out the possibility that the observed effects partly reflect demand characteristics—participants in the high-dependence condition may have reduced their effort because they were explicitly told to adopt AI outputs, rather than because they experienced genuine cognitive dependence. Future factorial experiments should disentangle these components. Third, the thinking orientation manipulation in Study 3 employed a brief situational priming procedure that induced a temporary, context-dependent mindset rather than a stable dispositional orientation. The effects of such short-term primes on creative performance may decay rapidly once the immediate experimental context is removed. Future research should therefore examine whether longer-term, curriculum-embedded interventions (e.g., process-oriented scaffolding across a full semester; reflective journaling protocols integrated with AI-assisted assignments) can produce durable shifts in thinking orientation. Fourth, all creativity tasks employed the Alternative Uses Task (AUT); although this confers high internal validity, ecological validity is limited. Future research should test the present findings in task contexts closer to authentic academic practice, such as academic paper conceptualisation, research proposal design, or essay writing. Fifth, although the samples spanned multiple disciplines, all participants were drawn from a single institution, and generalisability awaits cross-institutional verification. Future studies should replicate the experimental paradigm in multiple universities across different educational systems and cultural contexts. Sixth, the study focused on university students, but the pattern of generative AI’s impact may differ for primary and secondary school students or workplace professionals, warranting future investigation. Seventh, the experimental findings may be subject to demand characteristics: participants in the high-dependence condition may have inferred that the study aimed to show negative effects of AI and consequently reduced their creative effort. While the use of a cover story (“creative competition”) and the inclusion of a manipulation check partially mitigate this concern, future research could employ more subtle behavioural measures (e.g., implicit creativity tasks) that are less susceptible to demand effects. Eighth, the quality of the AI-generated outputs shown to participants may have influenced the results. If the AI-generated ideas were of lower quality than participants’ own ideas, the observed creativity deficits in the high-dependence condition could reflect output quality rather than dependence per se. We did not systematically evaluate the quality of AI outputs, and future studies should control for this. Ninth, the present findings are based on short-term, single-session experimental procedures. We cannot determine whether repeated exposure to high-dependence AI use would produce cumulative effects on critical thinking and creativity over weeks or months. Longitudinal field studies or semester-long classroom interventions would be needed to assess the long-term behavioural consequences. Tenth, the study relied on a specific student population from a single country; cultural attitudes toward AI, academic norms, and the prevalence of generative AI tools may differ substantially in other contexts, limiting the generalisability of the findings beyond the studied population.

## 6. Conclusions

(1) Generative AI dependence exerts a significant negative impact on university students’ creativity; this effect has been experimentally demonstrated.
(2) Critical thinking is the primary mediating pathway in the effect of AI dependence on creativity—AI dependence indirectly impairs creativity by weakening individuals’ critical thinking, with this mediation pathway supported at both correlational and experimental levels.
(3) Thinking orientation serves as an important boundary condition for the above mediation pathway: an outcome-oriented thinking style activates and amplifies the indirect impairing pathway from AI dependence to creativity via critical thinking, whereas a process-oriented thinking style effectively blocks this pathway by protecting critical thinking.

In sum, within the context of the Alternative Uses Task and under the instructional conditions employed here, AI tools per se are not an inherent threat to creativity—the risk lies in individuals’ loss of critical thinking engagement during the process of AI use. We caution against overgeneralising this conclusion: different pedagogical designs of AI integration (e.g., AI as a brainstorming partner with mandatory critique phases) may well produce neutral or even positive effects on creativity (see Boussioux et al., 2024; Chandrasekera et al., 2024). The present findings should therefore be interpreted as identifying one specific risk pathway—namely, unchecked dependence coupled with outcome-oriented thinking—rather than as a universal indictment of AI-assisted creativity. The key to education is not to keep students away from AI but to help them maintain the capacity for independent thinking, deep processing, and active creation in AI-assisted environments; cultivating a process-oriented approach to AI use is an effective pathway toward this goal.

## Figures and Tables

**Table 1:** Descriptive statistics and correlations among study variables in Study 1.

Variable	M	SD	1	2	3
1. AI dependence	3.42	0.89	—		
2. Critical thinking	3.15	0.42	−0.44 ***	—	
3. Creativity	3.68	0.76	−0.20 *	0.50 ***	—

Note. * *p* < 0.05, *** *p* < 0.001.

**Table 2:** Descriptive statistics and independent-samples *t*-tests for Study 2.

Variable	High AI Dependence	Low AI Dependence	*t*(130)	*p*	Cohen’s *d*
Manipulation check	4.39 (0.84)	3.82 (1.07)	−3.45	0.001	−0.60
Creativity	4.78 (0.66)	5.20 (0.53)	4.03	<0.001	0.70
Critical thinking	2.78 (0.29)	3.17 (0.28)	7.94	<0.001	1.37
Self-efficacy	3.45 (0.72)	3.52 (0.68)	0.58	0.566	0.10

Note. Standard deviations are reported in parentheses.

**Table 3:** Hierarchical moderated regression predicting critical thinking (Study 3).

Variable	Critical Thinking					
	Model 1		Model 2		Model 3	
	B	*SE*	B	*SE*	B	*SE*
Gender	0.07	0.07	0.03	0.07	0.03	0.07
Year	−0.07 *	0.03	−0.07 *	0.03	−0.07 *	0.03
AI dependence			−0.10	0.07	−0.26 **	0.09
Outcome/Process Orientation			0.28 ***	0.07	0.11*	0.09
AI dependence × thinking orientation					0.34 **	0.13
*R* ^2^	0.05		0.15		0.19	
Δ*R*^2^	0.05		0.10 ***		0.04 **	

Note. N = 160. * *p* < 0.05, ** *p* < 0.01, *** *p* < 0.001. Unstandardized coefficients (B) are reported.

**Table 4:** Hierarchical moderated regression predicting creativity (Study 3).

Variable	Creativity					
	Model 1		Model 2		Model 3	
	B	*SE*	B	*SE*	B	*SE*
Gender	0.22	0.18	0.11	0.10	0.12	0.10
Year	−0.02	0.08	−0.01	0.04	−0.01	0.03
AI dependence			−1.35 ***	0.09	−1.54 **	0.13
Outcome/Process Orientation			1.20 ***	0.09	1.00 ***	0.13
AI dependence × thinking orientation					0.39 *	0.18
*R* ^2^	0.04		0.70		0.72	
Δ*R*^2^	0.04		0.68 ***		0.01 *	

Note. N = 160. * *p* < 0.05, ** *p* < 0.01, *** *p* < 0.001. Unstandardized coefficients (B) are reported.

**Table 5:** Conditional indirect effects of the moderated mediation model in Study 3.

Thinking Orientation Condition	Indirect Effect	Boot SE	Bootstrap 95% CI
Outcome-oriented (W = 0)	−0.18	0.07	[−0.33, −0.05]
Process-oriented (W = 1)	0.05	0.06	[−0.07, 0.17]
Index of moderated mediation	0.22	0.10	[0.05, 0.43]

Note. Bootstrap = 5000 resamples.

## Data Availability

The original data presented in the study are openly available in the Psychological Data Bank at: https://cstr.cn/31253.11.sciencedb.psych.01038 (accessed on 15 July 2026); https://doi.org/10.57760/sciencedb.psych.01038 (accessed on 15 July 2026).

## Supplementary Materials

The following supporting information can be downloaded at: https://www.mdpi.com/article/10.3390/jintelligence14080168/s1, Table S1: Generative AI Dependence Scale; Table S2: Self-Report Creativity Scale; Table S3: Chinese Critical Thinking Structure Scale; Table S4: AI Creativity Self-Efficacy Scale; Table S5: Outcome-Oriented Priming Statements; Table S6: Process-Oriented Priming Statements; Table S7: Thinking Orientation Manipulation Check Items; Table S8: Consensual Assessment Technique (CAT) Scoring Rubrics; experimental task instructions for Studies 2 and 3.

## Abbreviations

The following abbreviations are used in this manuscript:

AI	Artificial Intelligence
ANOVA	Analysis of Variance
AUT	Alternative Uses Task
CAT	Consensual Assessment Technique
CMV	Common Method Variance
ICC	Intraclass Correlation Coefficient
IMM	Index of Moderated Mediation
