# Supplementary material for: The Impact of Generative AI Dependence on University Students’ Creativity: The Mediating Role of Critical Thinking and the Moderating Role of Thinking Orientation
Source: J Intell. 2026 Aug 1;14(8):168. doi: 10.3390/jintelligence14080168 (PMC13514692; doi:10.3390/jintelligence14080168)
Supplement: Supplementary file 1 [file jintelligence-14-00168-s001.zip › jintelligence-4392378-supplementary.pdf]

Supplementary Materials

Scale Items and Experimental Materials

This document provides the complete items for all scales used in the present research, as well as the experimental materials for Studies 2 and 3.

Table of Contents

1. Generative AI Dependence Scale;
2. Self-Report Creativity Scale;
3. Chinese Critical Thinking Structure Scale;
4. AI Creativity Self-Efficacy Scale;
5. Thinking Orientation Priming Materials (Study 3);
6. Thinking Orientation Manipulation Check (Study 3);
7. Experimental Task Instructions (Studies 2 and 3).

1. Generative AI Dependence Scale

Source: Adapted from Tang et al. (2023) for the student context.

Original source: Tang, P. M., Koopman, J., Yam, K. C., De Cremer, D., Zhang, J. H., & Reynders, P. (2023). The self-regulatory consequences of dependence on intelligent machines at work. *Human Resource Management,* 62(5), 721–744.

Response scale: 1 = strongly disagree, 5 = strongly agree.

Table S1. Generative AI Dependence Scale.

Item No.	Item Text (Chinese)	Item Text (English)
1	我依赖生成式 AI 来处理或协助学术研究/学习任务。	I depend on generative AI to handle or assist with academic research/study tasks.
2	我依赖生成式 AI 来制定重要的研究决策。	I depend on generative AI to make important research decisions.
3	我依赖生成式 AI 来检查和监控我的学习/研究成果的准确性。	I depend on generative AI to check and monitor the accuracy of my learning/research outcomes.

Note: ***The original scale was developed in a workplace context. For the present study, work-related referents (e.g., "work tasks") were replaced with academic equivalents (e.g., "academic research/study tasks"). All items retained their original meaning and structure.***

Psychometric properties in the present sample (Study 1): α = 0.79.

2. Self-Report Creativity Scale

Source: Adapted from Akpur (2020).

Original source: Akpur, U. (2020). Critical, reflective, creative thinking and their reflections on academic achievement. Thinking Skills and Creativity, 37, 100683.

Response scale: 1 = almost never, 5 = always.

Table S2. Self-Report Creativity Scale.

Item No.	Item Text (Chinese)	Item Text (English)
1	我能够提出具有原创性且具有现实意义的研究问题。	I am able to propose research questions that are both original and practically meaningful.
2	我能够从新的视角解读研究问题，提供不同的见解。	I am able to interpret research problems from new perspectives and offer different insights.
3	我能够发明或巧妙运用研究技术、研究工具或实验设备。	I am able to invent or skillfully apply research techniques, tools, or experimental equipment.
4	我能够提出突破研究困境的新路径、新方法、新流程或新手段。	I am able to propose new paths, methods, processes, or means to break through research difficulties.
5	在研究过程中，我能够发现并提供新的证据来回答研究问题。	In the research process, I am able to discover and provide new evidence to answer research questions.
6	我能够归纳研究发现，并提出可推广的新理论。	I am able to summarise research findings and propose generalisable new theories.

Note: This scale was originally designed to measure general creative thinking capacity. In the present study, the items were framed within an academic/research context to align with the student sample. The scale captures self-perceived creative capacity across domains rather than performance on a specific creative task.

Psychometric properties in the present sample (Study 1): α = 0.87.

3. Chinese Critical Thinking Structure Scale

Source: Hou et al. (2022).

Original source: Hou, Y., Li, Q., & Li, H. (2022). Construction of the Chinese critical thinking structure scale and its psychometric properties. *Acta Scientiarum Naturalium Universitatis Pekinensis,* 58(2), 383–390.

Response scale: 1 = strongly disagree, 5 = strongly agree.

Table S3. Chinese Critical Thinking Structure Scale.

Item No.	Dimension	Item Text (Chinese)	Item Text (English)
1	Analyticity	做事情之前，我清楚地知道自己的目的是什么。	Before doing things, I clearly know what my purpose is.
2	Systematicity	我喜欢按照一定的步骤去解决问题。	I like to solve problems by following certain steps.
3	Open-mindedness	我做决策时经常考虑父母长辈的意见。	I often consider the opinions of my parents and elders when making decisions.
4	Analyticity	面对问题时，我会根据不同的情境做出不同的决定。	When facing problems, I make different decisions based on different situations.
5	Analyticity	我能够区分生活中哪些事情对我当前是重要的。	I am able to distinguish which things in life are important to me at present.
6	Open-mindedness	除了与课程有关的阅读外，我也会阅读很多课外的东西。	In addition to course-related reading, I also read a lot of extracurricular material.
7	Open-mindedness	我会仔细考虑"存在必有其合理性"这句话的可靠性。	I carefully consider the validity of the statement "Existence implies rationality."
8	Open-mindedness	我可以与拥有不同观点的人进行交流。	I can communicate with people who hold different views.
9	Attention check	此题请选择"完全不同意"。	Please select "strongly disagree" for this item.
10	Open-mindedness	我不能容忍别人不同意我的观点或想法。	I cannot tolerate others disagreeing with my views or

			ideas. (Reverse-scored)
11	Open-mindedness	中途改变自己的主意是很丢脸的事情。	Changing one's mind halfway is a shameful thing. (Reverse-scored)
12	Systematicity	做事时，除了运用当前的方法，我不会再考虑其他方法。	When doing things, besides using the current method, I will not consider other methods. (Reverse-scored)
13	Open-mindedness	与别人产生意见分歧会影响到我们之间的交流。	Having disagreements with others will affect our communication. (Reverse-scored)
14	Systematicity	面对复杂问题时，我常常无从下手。	When facing complex problems, I often do not know what to do. (Reverse-scored)
15	Open-mindedness	我经常与别人分享我对事物的看法。	I often share my views on things with others.
16	Systematicity	看到一个新产品的说明书复杂难懂时，我倾向于直接进行实际操作。	When I see a new product manual that is complex and difficult to understand, I tend to operate it directly.
17	Systematicity	我解决问题时经常会有替代方案。	I often have alternative solutions when solving problems.
18	Systematicity	同龄人遇到难以决断的问题时，喜欢找我商量。	When peers encounter difficult-to-decide problems, they like to consult with me.

Note: Item 9 is an attention-check item and was excluded from scale scoring. Items 10–14 are reverse-scored. The scale comprises three dimensions: analyticity (Items 1, 4, 5), systematicity (Items 2, 12, 14, 16, 17, 18), and open-mindedness (Items 3, 6, 7, 8, 10, 11, 13, 15). In the present study, all 17 substantive items (excluding the attention check) were used as a composite critical thinking index.

Psychometric properties in the present sample (Study 1): α = 0.85.

4. AI Creativity Self-Efficacy Scale

Source: Adapted from Wang & Chuang (2024).

Original source: Wang, Y.-Y., & Chuang, Y.-W. (2024). Artificial intelligence self-efficacy: Scale development and validation. *Education and Information Technologies*, 29(4), 4785–4808.

Response scale: 1 = strongly disagree, 5 = strongly agree.

Table S4. AI Creativity Self-Efficacy Scale.

Item No.	Item Text (Chinese)	Item Text (English)
1	我有信心在不依赖 AI 的情况下生成有创意的想法。	I am confident that I can generate creative ideas without relying on AI.
2	即使不依赖 AI，我也能够提出独特的创意。	Even without relying on AI, I am able to propose unique creative ideas.
3	在独立完成创造性任务时，我对自己的创意生成能力充满信心。	When independently completing creative tasks, I am fully confident in my creative generation ability.

Note: This scale was used as an alternative mediator in Study 2 to test whether the effect of the AI-dependence manipulation on creativity was better explained by changes in task confidence rather than critical thinking. The mediating effect of self-efficacy was non-significant (indirect effect = −0.12, 95% CI = [−0.40, 0.16]).

Psychometric properties in the present sample (Study 2): α = 0.76.

5. Thinking Orientation Priming Materials (Study 3)

Source: Adapted from Dai et al. (2023).

Original source: Dai, W., Yang, T., White, B. X., Palmer, R., Sanders, E. K., McDonald, J. A., Leung, M., & Albarracín, D. (2023). Priming behavior: A meta-analysis of the effects of behavioral and nonbehavioral primes on overt behavioral outcomes. *Psychological Bulletin,* 149(1-2), 67–98.

Note: The priming procedure was designed to induce a temporary, situation-specific thinking orientation rather than to measure or alter a stable, dispositional trait. Participants in each condition read and rated five biased statements on a 7-point scale (1 = very unimportant, 7 = very important).

5.1 Outcome-Oriented Priming Statements

Table S5. Outcome-Oriented Priming Statements.

No.	Statement (Chinese)	Statement (English)
1	学习的结果必须是有回报的。	The outcome of learning must be rewarding.
2	我关心我从学习项目中最终获得了什么。	I care about what I ultimately gain from a learning project.
3	从项目中获得成果是非常重要的。	Obtaining results from a project is very important.
4	学习的结果本身具有很高的价值。	The outcome of learning itself has high value.
5	一个好的结果比过程中的努力更重要。	A good result is more important than effort in the process.

5.2 Process-Oriented Priming Statements

Table S6. Process-Oriented Priming Statements.

No.	Statement (Chinese)	Statement (English)
1	学习的过程必须是愉快的。	The process of learning must be enjoyable.
2	我关心我在学习项目中的体验。	I care about my experience in a learning project.
3	学习的过程对我来说非常重要。	The process of learning is very important to me.
4	学习的过程本身具有价值。	The process of learning itself has value.
5	学习过程中的学习和成长比最终结果更有价值。	Learning and growth in the process are more valuable than the final result.

6. Thinking Orientation Manipulation Check (Study 3)

Participants rated the following two items on a 7-point Likert scale to verify the effectiveness of the thinking orientation priming.

Table S7. Thinking Orientation Manipulation Check Items.

No.	Item Text (Chinese)	Item Text (English)	Anchors
1	与学习结果相比，我认为学习过程更重要 / 学习结果更重要	Compared with learning outcomes, I think the learning process is more important / learning outcomes are more important	1 = process more important, 7 = outcomes more important
2	与最终成果相比，我更在意学习过程中的体验 / 我更在意最终成果的达成	Compared with the final outcome, I care more about the experience during the learning process / I	1 = process-oriented, 7 = outcome-oriented

		care more about achieving the final outcome	

Manipulation check results: The outcome-oriented group scored significantly higher (M = 4.53, SD = 1.77) than the process-oriented group (M = 3.61, SD = 1.87), t(158) = 3.18, p = 0.002, Cohen's d = 0.50.

7. Experimental Task Instructions (Studies 2 and 3)

7.1 Study 2: AI Dependence Manipulation

Phase 1: Priming Task

High-dependence group instructions:

阶段一：您需要使用 AI 来完成几个简单任务。您需要使用 AI 提供的答案（请直接抄写 AI 结果）。请不要进行额外计算或尝试自己解答，AI 提供的答案是最优的。

(Phase 1: You need to use AI to complete several simple tasks. You need to use the answers provided by AI (please copy the AI results directly). Please do not perform additional calculations or attempt to solve on your own; the answers provided by AI are optimal.)

Low-dependence group instructions:

阶段一：您需要完成几个简单任务。请自主思考，不要使用外部工具。

(Phase 1: You need to complete several simple tasks. Please think independently and do not use external tools.)

Task 1 (Mathematical calculation): 57 × 83 = ______

Task 2 (Text summarisation): Read the following short text and summarise the core content.

"Coral reefs are important components of marine ecosystems, providing habitats for millions of marine species. Corals obtain energy through cooperation with symbiotic algae, but global warming causes sea temperature rise, leading to coral bleaching. Research shows that sustained ocean warming may cause more than 90% of coral reefs to disappear by the end of this century. Scientists are studying the genes of coral heat resistance in hopes of cultivating more heat-tolerant coral species."

Task 3 (English–Chinese translation): Translate the following English text into Chinese.

"Weather changes throughout the year. In summer, the sun is strong, and temperatures are high. In winter, cold air brings snow and ice. Rainy days make the air feel cooler, and the sky looks

gray. Strong winds can move clouds and sometimes cause storms. Weather affects daily life, from the clothes people wear to how they travel."

Phase 2: Creativity Task (Alternative Uses Task)

Common instructions to all participants:

您将参与一个创意竞赛，目标是为特定情境提出一个有创意的想法！

(You will participate in a creative competition, aiming to propose a creative idea for a specific situation.)

High-dependence group (creativity task):

您必须使用 AI（ChatGPT）来生成您的创意，且尽量不要修改 AI 生成的答案。

(You must use AI (ChatGPT) to generate your creative idea, and try not to modify the AI-generated answer.)

Low-dependence group (creativity task):

您可以使用 AI（ChatGPT）来生成您的创意，并可以自由修改 AI 生成的内容。

(You may use AI (ChatGPT) to generate your creative idea, and you are free to modify the AI-generated content.)

Creative prompt: Imagine you are cleaning out your garage and find two unused items: a tennis racket and a garden hose. You decide to be creative and repurpose these items, giving them new uses. Please come up with 1 innovative and practical method within 10 minutes to combine these two items and create a new use.

7.2 Study 3: AI Dependence Manipulation (Trust Game Paradigm)

Source: Adapted from Klingbeil et al. (2024).

Original source: Klingbeil, A., Grützner, C., & Schreck, P. (2024). Trust and reliance on AI — An experimental study on the extent and costs of overreliance on AI. Computers in Human Behavior, 160, 108352.

Task introduction:

接下来你将与另一位玩家 B 进行一个博弈任务：

你和玩家 B 各自拥有 10 个积分。你需要决定是否选择与对方"合作（IN）"或"拒绝合作（OUT）"。

如果你选择 OUT，你和对方各自保留 10 积分。

如果你选择 IN，则进入博弈阶段：

如果对方也选择 IN：你和对方都将获得 20 积分（共赢）；

如果对方选择 OUT：你获得 0 积分，对方获得 40 积分（你损失）；

此轮结束后，你将看到对方的选择结果。

(Next, you will play a game with another Player B: You and Player B each have 10 points. You need to decide whether to choose "cooperate (IN)" or "refuse to cooperate (OUT)". If you choose OUT, you and the other player each keep 10 points. If you choose IN, the game proceeds: If the other player also chooses IN: both you and the other player receive 20 points (win-win); If the other player chooses OUT: you receive 0 points, the other player receives 40 points (you lose). After this round, you will see the other player's choice.)

Round 1: Player B chooses IN.

Round 2: Player B chooses OUT.

Round 3 (critical manipulation round):

High-AI-advice group:

以下是由人工智能（AI）助手根据该玩家以往行为生成的决策建议：

"根据玩家 B 过去的行为模式，尽管其存在一次不合作行为，但其也表现出一定的合作意愿。根据 AI 系统的综合分析，建议你在本轮选择合作（IN），以争取潜在的双赢结果。"

请你根据历史信息和 AI 建议，自行做出本轮决策。

(The following is a decision suggestion generated by an artificial intelligence (AI) assistant based on the player's past behavior: "According to Player B's past behavioural patterns, although there was one instance of non-cooperation, they have also shown a certain willingness to cooperate. Based on the comprehensive analysis of the AI system, it is recommended that you choose to cooperate (IN) in this round to strive for a potential win-win outcome." Please make your own decision for this round based on historical information and AI advice.)

No-AI-advice group (low-dependence):

请你参考以下这位玩家 B 的历史行为记录，自主判断对方是否值得信任，并做出本轮决策。

(Please refer to the following historical behaviour record of Player B, independently judge whether the other party is trustworthy, and make your decision for this round.)

Manipulation check:

我在做出选择时主要依赖了自己独立的判断。(反向题)

(I mainly relied on my own independent judgment when making the choice. [Reverse-scored])

Response scale: 1 = strongly disagree, 7 = strongly agree.

7.3 Study 3: Creativity Task

Task instructions (all conditions):

您将参与一个创意竞赛，目标是为特定情境提出一个有创意的想法！

假设你现在手头有三个不再使用的物品：

一个纸袋

一堆建筑剩余的砖块

一个未使用的风扇

请结合你所学专业的知识和技能，设计一个创新且实用的物品或工具，以帮助解决现实中的某个具体问题。

请在 10 分钟内想出 1 个创新且实用的方法，将这三样物品结合在一起，创造新的用途。

(You will participate in a creative competition, aiming to propose a creative idea for a specific situation! Assume you now have three unused items on hand: a paper bag, a pile of leftover construction bricks, and an unused fan. Please use the knowledge and skills from your major to design an innovative and practical item or tool to help solve a specific real-world problem. Please come up with 1 innovative and practical method within 10 minutes to combine these three items and create a new use.)

High-dependence group:

您必须使用 AI（ChatGPT）来生成您的创意，且尽量不要修改 AI 生成的答案。

(You must use AI (ChatGPT) to generate your creative idea, and try not to modify the AI-generated answer.)

Low-dependence group:

您可以使用 AI（ChatGPT）来生成您的创意，并可以自由修改 AI 生成的内容。

(You may use AI (ChatGPT) to generate your creative idea, and you are free to modify the AI-generated content.)

Consensual Assessment Technique (CAT) Scoring Rubrics

Creativity was rated by three trained independent raters on three dimensions using a 7-point Likert scale.

Table S8. Consensual Assessment Technique (CAT) Scoring Rubrics.

Dimension	Definition	Anchor 1 (not at all)	Anchor 7 (extremely)
Novelty	Originality and uncommonness of the response	Very common, mundane uses	Highly unusual, unexpected uses
Appropriateness	Feasibility and usefulness of the response	Completely impractical or nonsensical	Highly practical and sensible
Fluency	Quantity and diversity of responses	Very few or repetitive uses	Numerous and varied uses

Inter-rater reliability (ICC, two-way random, absolute agreement, single measures): novelty ICC = 0.88, appropriateness ICC = 0.84, fluency ICC = 0.85; overall ICC = 0.86.

End of Supplementary Materials
